# Establishment of an Immortalized Skin Keratinocyte Cell Line Derived from the Animal Model *Mastomys coucha*

**DOI:** 10.1371/journal.pone.0161283

**Published:** 2016-08-17

**Authors:** Daniel Hasche, Sonja Stephan, Larissa Savelyeva, Frank Westermann, Frank Rösl, Sabrina E. Vinzón

**Affiliations:** 1 Division of Viral Transformation Mechanisms (F030), German Cancer Research Center, Heidelberg, Germany; 2 Division of Neuroblastoma Genomics (B087), German Cancer Research Center, Heidelberg, Germany; University of Alabama at Birmingham, UNITED STATES

## Abstract

In the present report we describe the establishment of a spontaneous immortalized skin keratinocyte cell line derived from the skin of the multimammate rodent *Mastomys coucha*. These animals are used in preclinical studies for a variety of human diseases such as infections with nematodes, bacteria and papillomaviruses, especially regarding cutaneous manifestations such as non-melanoma skin cancer. Here we characterize the cells in terms of their origin and cytogenetic features. Searching for genomic signatures, a spontaneous mutation in the splicing donor sequence of *Trp53* (G to A transition at the first position of intron 7) could be detected. This point mutation leads to alternative splicing and to a premature stop codon, resulting in a truncated and, in turn, undetectable form of p53, probably contributing to the process of immortalization. *Mastomys coucha*-derived skin keratinocytes can be used as an *in vitro* system to investigate molecular and immunological aspects of infectious agent interactions with their host cells.

## Introduction

The multimammate rodent *Mastomys* [1] serves as a suitable model for diseases caused by numerous infectious agents such as Brugia malayi [2], Trypanosoma [3], Helicobacter pylori [4], Lassa fever virus [5] and papillomaviruses [6, 7]. *In vivo* models allow the dissection of infection routes, to study cancer development and to test the efficacy of vaccination against the respective infectious agent [8–10]. In our previous studies, we have used *Mastomys coucha* as a model to study the role of cutaneous papillomaviruses and their function in the context of non-melanoma skin cancer [11, 12].

The animals housed at the German Cancer Research Center (DKFZ) are persistently infected with the *Mastomys natalensis* papillomavirus (MnPV) and *Mastomys coucha* papillomavirus 2 (McPV2) [7] and spontaneously develop epithelial lesions like warts, keratoacanthomas and squamous cell carcinomas linked to MnPV [11]. We previously showed that the development of skin tumors in these animals can be efficiently prevented by prophylactic vaccination based on virus-like particles (VLP) even under immunosuppressive conditions [11]. Moreover, we recently reported the complete MnPV transcription map derived from productive lesions in animals and found homologous transcripts known from HPVs as well as novel splicing isoforms for proteins of unknown function [13].

Although animal models are essential to mimic a clinical scenario seen in patients, it is also necessary to design reductionist molecular approaches under *in vitro* conditions, using a homogeneous population of cells to study the bidirectional cross-talk between virus and host, thereby making *Mastomys coucha*-derived keratinocytes extremely desirable, especially considering the strict species-specificity in the case of papillomavirus infection. Here, we describe the establishment and characterization of such cells (referred as Kera5) obtained from the skin of virus-free animals.

## Methods

### Animals

*Mastomys coucha* from the DKFZ breeding colony were maintained under standard conditions in compliance with German and European statutes [11] and all experiments were undertaken with the approval of the responsible Animal Ethics Committee (Regional Council of Karlsruhe, Germany; G26/12, DKFZ 276). Virus-free animals were obtained by hysterectomies of pregnant *Mastomys* under sterile conditions [11]. The offspring were nursed by foster specified pathogen-free (SPF) mice (*Mus musculus*), kept in a specific pathogen free isolator unit at the DKFZ.

### Isolation of skin keratinocytes and fibroblasts

*Mastomys* keratinocytes were isolated as described [14, 15]. Briefly, newborn animals were sacrificed by decapitation and carcasses were disinfected by submersion in iodine solution (5 min) and 70% ethanol (5 min) prior to removal of extremities under aseptic conditions. A longitudinal incision was made from neck to tail and the skin was peeled off. Skins were allowed to float two times (10 min) in gentamycin (0.25 mg/ml in PBS) and were spread out in a petri dish and incubated overnight at 4°C with 5 mg/ml Dispase II (Roche) in dKSFM (Thermo Fisher Scientific) to separate epidermis and dermis. The epidermis was peeled off the dermis and incubated with 1.25% trypsin (Sigma-Aldrich) in PBS (20 min at room temperature) to separate the keratinocytes. To favor the process, the epidermis was ripped with forceps. Trypsinization was stopped by addition of defined Keratinocyte-SFM (dKSFM, Thermo Fisher Scientific) supplemented with 10% FCS (Thermo Fisher Scientific). The suspension was filtered through a 70 μm cell strainer (Falcon) and centrifuged for 5 min at 400xg. The pellet was resuspended in conditioned dKSFM obtained from *Mastomys*-derived fibroblasts, supplemented with penicillin/streptomycin (Thermo Fisher Scientific) and 1.15 x 10^5^ cells were seeded per 6 cm dish. Medium was changed every two days.

In order to establish *Mastomys*-derived fibroblasts for the production of conditioned keratinocyte medium, the dermis was cut into pieces, spread in a cell culture dish and air dried for 15 min in the cell culture hood prior to the addition of DMEM supplemented with 4.5 g/l glucose, 20% FSC, 2 mM L-Gln, 100 U penicillin and 100 μg/l streptomycin (Thermo Fisher Scientific). The medium was changed every two days. Outgrowing fibroblasts were collected by trypsinization with 0.25% trypsin/EDTA (Thermo Fisher Scientific) and filtered through a 70 μm cell strainer. The cells were subsequently cultured in DMEM supplemented with 10% FCS.

### Cell culture

Kera5 were cultivated in conditioned dKSFM supplemented with 1 nM cholera toxin, 5 ng/ml human EGF and supplements provided by the manufacturer. During the first ten passages, 10 μM Rho kinase inhibitor Y-27632 (R&D systems) was added to the medium to favor proliferation [16]. Kera5 were split at a confluence of 70–80% by washing with PBS and trypsinizing with 0.25% trypsin/EDTA. The reaction was stopped with trypsin inhibitor from soya beans (Sigma-Aldrich). Cells were centrifuged at 350xg for 3 min. The pellet was resuspended and seeded at a confluence of 30–50%. Murine NIH 3T3 fibroblasts, H1299 cells and *Mastomys*-derived fibroblast cell line MaFi132 were cultivated in DMEM supplemented with 4.5 g/l glucose, 10% FSC and 2 mM L-Gln. For passaging, the cells were washed with PBS and trypsinized with 0.25% trypsin/EDTA. The reaction was stopped by adding DMEM supplemented with 10% FCS. Cells were centrifuged at 350xg for 3 min, resuspended and split up to 1:20. Conditioned medium (CM) was produced by cultivation of MaFi132 cells for 48 h in dKSFM. Since MaFi132 cells had the tendency to detach in dKSFM, the medium was filtered before Kera5 cells were grown in CM that was supplemented with one part of fresh dKSFM. Murine 308 keratinocytes [17] were cultivated in MEM medium supplemented with 10% FCS.

### Immunofluorescence stainings

Kera5 were seeded on glass cover slides in dKSFM. 24 h later, cells were washed with PBS and fixed for 10 min with ice cold acetone. To induce differentiation, cells were further incubated for 24 h in dKSFM supplemented with additional calcium (PromoCell) prior to the fixation, as indicated in the figure legend. Cells were blocked in 1% BSA/0.3% Triton X-100 in PBS for 1 h and stained with specific antibodies against keratin 14 (Covance PRB-155P), vimentin (D21H3; CST #5741) or involucrin (Covance PRB-140C) and the respective secondary goat anti-rabbit IgG (conjugated to AlexaFluor488 or AlexaFluor594, Invitrogen). Nuclei were stained with DAPI. Cover slides were mounted with Faramount Aqueous Mounting Medium (Dako) and imaged with a BZ-9000 fluorescence microscope (Keyence).

### Isolation of splenocytes and analysis of metaphases

Splenocytes were extracted from *Mastomys coucha* by mashing the spleen through a 100 μm cell strainer (Falcon) into DMEM-10 (DMEM supplemented with 10% FCS and 2 mM L-Gln), centrifuged for 5 min at 800xg and resuspended in DMEM-10. Splenocytes and Kera5 were incubated in 0.5 μg/ml KaryoMAX Colcemid (Thermo Fisher Scientific) diluted in DMEM-10 or dKSFM for 2 h at 37°C. Harvested cells were pelleted and treated with hypotonic solution (1% NaCl and 0.55% KCl in H_2_O, 1:1) for 25 min prior to fixation with methanol-acetic acid (3:1). Cells were spread on microscope slides, stained with DAPI and imaged at 630x magnification with an Imager.Z1 fluorescence microscope (Carl Zeiss). At least 12 metaphases were analyzed per sample using Meta Systems ISIS software (Carl Zeiss).

### Preparation of nucleic acids

Genomic DNA was isolated using the QIAamp DNA Mini Kit (Qiagen). RNA was isolated using the RNeasy^®^ Mini Kit (Qiagen) according to the manufacturer’s protocol. Additional treatment with the TURBO DNA-free^™^ Kit (Ambion) was performed to eliminate traces of DNA. RNA integrity was assessed by visualization of sharp 28S and 18S rRNA bands on a 1% agarose gel.

### Reverse Transcription (RT) and Polymerase Chain Reaction (PCR)

Reverse Transcription (RT) was performed with the RevertAid Reverse Transcriptase. 1 μg of total RNA was incubated with 200 ng Oligo-dT_18_ primers and denatured at 65°C for 5 min. The RT reaction was carried out by adding 5X RT Buffer, 10 mM DTT, 0.5 mM dNTPs, 10 U RiboLock and 200 U RevertAid RT enzyme (all Thermo Fisher Scientific) and incubating at 42°C for 60 min, followed by a final heating at 70°C for 10 min to inactivate the enzyme. *Trp53* and p53 cDNA were amplified from genomic DNA or reverse transcribed RNA by PCR using PRECISOR High-Fidelity DNA Polymerase (BioCat) and appropriate primers (S1 Table) according to the manufacturer’s protocol. Thermal cycling conditions for PCRs were based on a primary denaturation step at 98°C for 2 min, followed by 37 cycles of 30 sec at 98°C, 20 sec at 58–60°C, 25 sec at 72°C and a final extension step of 5 min at 72°C.

### Gel purification and sequencing of PCR products

DNA fragments were separated according to their size by agarose gel electrophoresis, stained with 0.5 μg/mL ethidium bromide and visualized by UV light. As a size standard, GeneRuler^™^ 1 kb DNA ladder (Thermo Fisher Scientific) was run on the same gel. DNA from agarose gels was extracted with the QIAquick^®^ Gel Extraction Kit (Qiagen) according to the manufacturer’s protocol. DNA fragments were sequenced using the GATC Biotech Sanger Service (GATC Biotech, Konstanz). Chromatograms were analyzed with Chromas 2.5.3 (Technelysium).

### Cloning of expression vectors and transfection of MaFi132

The wildtype p53 (p53wt) coding sequence was amplified from cDNA obtained from freshly isolated keratinocytes using PRECISOR High-Fidelity DNA Polymerase with appropriate primers (S1 Table). The amplified DNA was cloned into the pPK-CMV-E3 expression vector (PromoKine) enabling expression of proteins tagged to HA (hemagglutinin). The truncated version of p53 (p53trunc) was amplified by PCR from the pPK- p53wt vector and also cloned into pPK-CMV-E3 using the BamHI and EcoRI restriction sites. The forward primer was the same one used for amplification of p53wt while the reverse primer was complementary to the last nucleotides at the 5´-end of the mutation. MaFi132 were transfected with 3 μg of the respective expression plasmids (p53wt or p53trunc) with Lipofectamine3000 (Thermo Fisher Scientific) according to the manufacturer’s protocol and harvested 24 h after transfection.

### Transactivation reporter assay

To show the functionality of *Mastomys* wildtype p53, 1x10^5^ H1299 cells/well were co-transfected with 50 ng pPK-p53 (p53wt) or the putative truncated version of p53, 400 ng pG13-luc encoding firefly luciferase under the control of the p53 consensus binding site of the p21 promoter [18] and 100 ng pRL-TATA encoding a TATA box-driven Renilla luciferase for normalization of the signals [19] using Lipofectamine3000 according to the manufacturer’s protocol. All transfections were performed in duplicates. 24 h after transfection, cells were harvested, lysed with 1x passive lysis buffer included in the Dual-Luciferase^®^ Reporter Assay System (Promega) and the activities of both luciferases (RLU, relative light units) in 20 μl lysate were measured according to the manufacturer’s protocol in a Synergy2 reader (BioTek). The transactivation activity of wildtype p53 was set to 100%. For statistical analysis a t-test was used.

### Cell damage and Western blotting

For UV irradiation, cells were washed once with PBS and irradiated with the respective dose of UVB (Waldmann UV181BL with an output range of 280 to 320 nm) as measured with a detector (Waldmann Variocontrol). The cells were further incubated with fresh medium. Incubation times and treatment with adriamycin (Sigma-Aldrich) was done as described in the figure legend. Cell lysates were prepared as described previously [20]. 25 μg (transfected MaFi132) or 50 μg (Kera5, NIH 3T3) of denatured cell lysate were loaded to SDS-PAGE. After blotting, proteins were detected with anti-p53 (Pab240, BD Biosciences) or an anti-mouse-actin antibody prior to detection with anti-mouse-HRP (Promega).

### Alignment

Murine *Trp53* (Gene ID 22059) and rat *Tp53* (Gene ID 24842) served as reference sequences for alignments with *Mastomys* sequences using Clustal 2.0.12.

## Results

### Establishment of *Mastomys*-derived keratinocyte cell line Kera5

**Fig 1:**
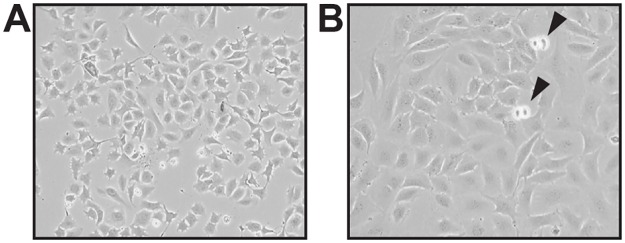
Cell morphology. **(A)** *Mastomys coucha* keratinocytes at passage 6. **(B)** Kera5 at passage 175, showing a typical cobblestone phenotype and an increased cell size (arrowheads mark ongoing mitoses; magnification: 200x).

**Fig 2:**
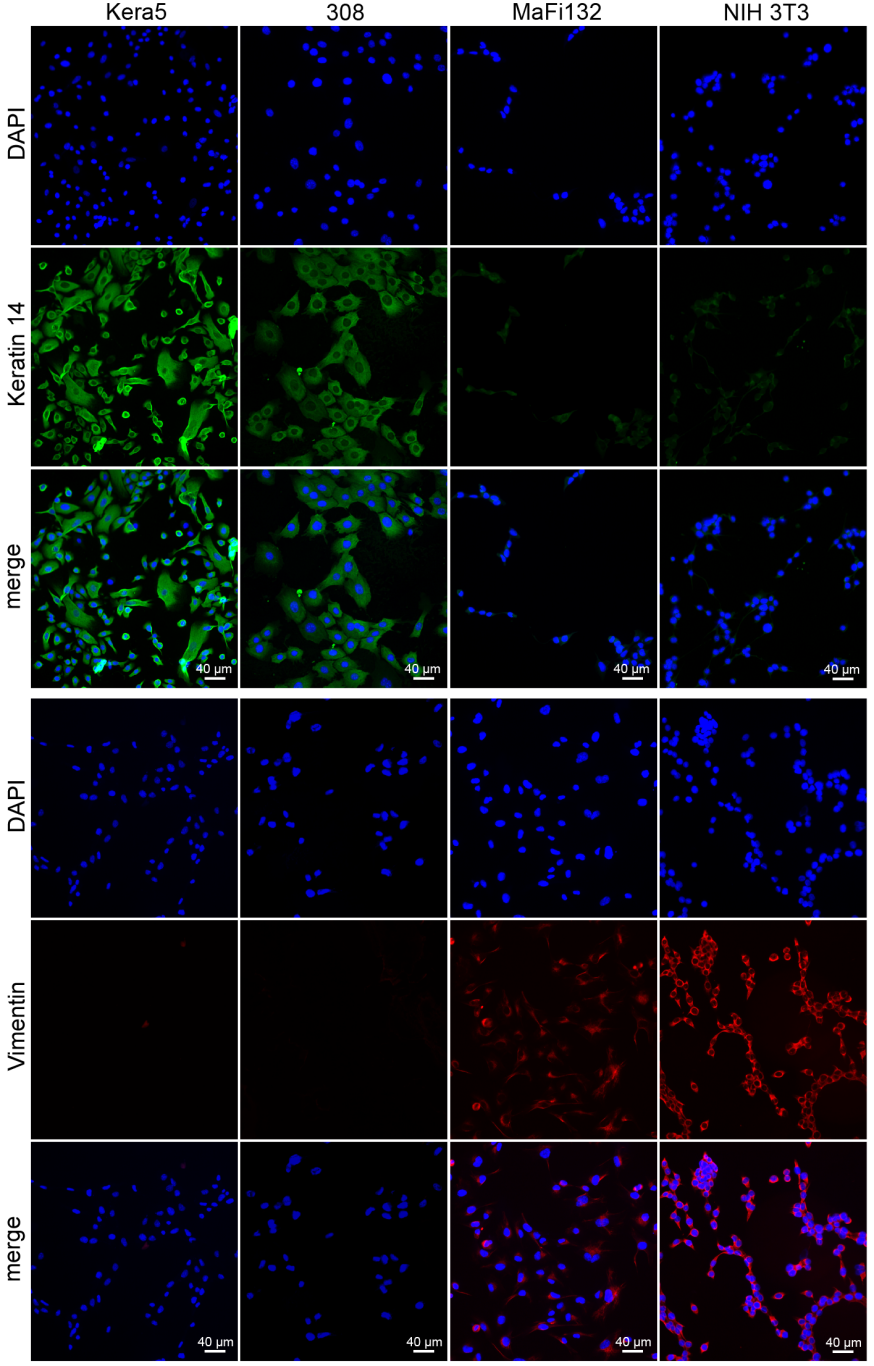
Indirect immunofluorescence. Kera5, MaFi132, 308 and NIH 3T3 cells were grown on cover slips, fixed with acetone, stained with keratin 14 or vimentin specific antibodies and detected with AlexaFluor488 or AlexaFluor594-conjugated secondary antibodies, respectively. Murine keratinocytes (308 cells) and fibroblasts (NIH 3T3) were used as controls. Green fluorescence indicates keratin 14 expression only in Kera5 and 308 cells, whereas red fluorescence shows vimentin expression only in MaFi132 and NIH 3T3 (original magnification: 200x).

To avoid the laborious work of repeated explantation of cells from tissue and to have a permanent cell line available for further *in vitro* studies, primary keratinocytes were isolated from the skin of newborn virus-free *Mastomys coucha*. In order to facilitate their immortalization, proliferation during the first ten passages was supported by addition of the Rho kinase inhibitor Y-27632 [16] and constant cultivation in conditioned medium obtained from *Mastomys*-derived fibroblast cell line MaFi132. This procedure finally led to the establishment of a keratinocyte cell line, referred as Kera5, which could be propagated now for more than 175 passages. Microscopic examination of these cells at both early and late passages with the same magnification shows a typical cobblestone appearance and an increased cell size (Fig 1, compare A and B) that may point to a change of the nuclear/cytoplasm ratio, indicative for polyploidy [21, 22]. In order to confirm their epidermal origin, immunofluorescence staining was performed. For this purpose, murine 308 keratinocytes obtained from 7,12-Dimethylbenz(a)anthracene (DMBA) treated mouse skin [17] were used as positive control. MaFi132 and murine NIH 3T3 fibroblasts served as negative staining controls. Fig 2 shows that similar to murine 308 cells, Kera5 highly expressed cytokeratin 14 as typical marker of basal keratinocytes [23]. Conversely, both Kera5 and 308 cells were negative for vimentin, a marker protein which is characteristic for cells of mesenchymal origin such as MaFi132 and NIH 3T3 fibroblasts [24].

### Karyotyping the Kera5 cell line

**Fig 3:**
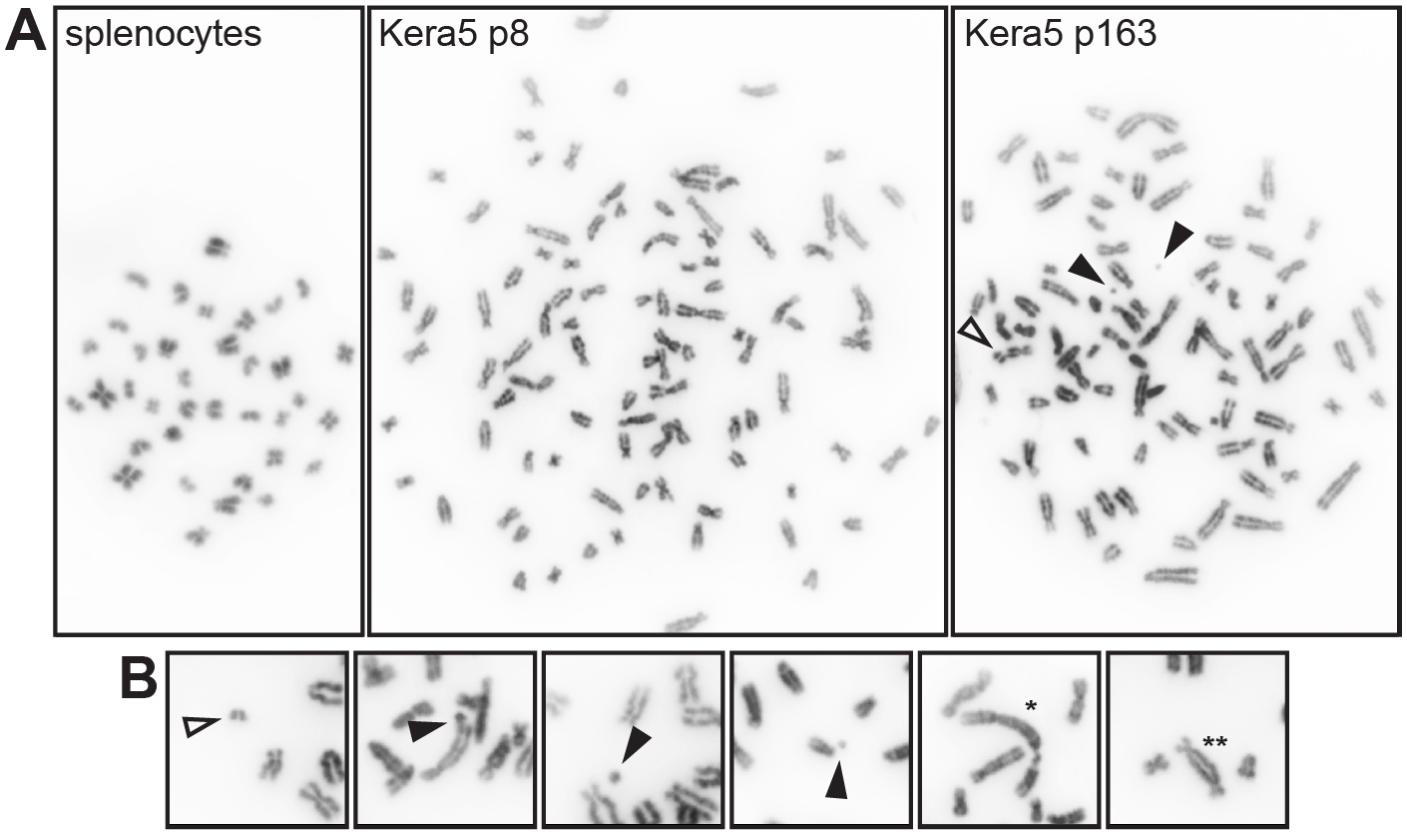
Karyotype analysis of Kera5 cells. **(A)** Representative metaphases of freshly isolated *Mastomys coucha* splenocytes in comparison with Kera5 at passage 8 and passage 163. Splenocytes are diploid (n = 36), whereas polyploidy can already be discerned at p8 (average n = 100; ranging from n = 92 to 111). The chromosome set in Kera5 at p163 is smaller (average n = 83; ranging from n = 78 to 86). **(B)** Chromosomal instability is further demonstrated by chromatid breaks and fragments (black arrowheads), acentric fragments (white arrowheads), rearrangements leading to marker chromosomes (*) and dicentric chromosomes (**). Metaphasic chromosomes were obtained by subsequent block with colcemid, hypotonic treatment and fixation in methanol-acetic acid. Cell suspensions were spread on microscope slides, stained with DAPI and imaged at 630x magnification.

Since Kera5 could be kept in culture for more than 165 passages without Rho kinase inhibitor, the cells are considered as spontaneously immortalized. In a next set of experiments, we analyzed the karyotype of early and late passage cells in comparison to freshly isolated splenocytes (Fig 3A). Splenocytes were used as reference cells because they do not require long adaptation for *in vitro* growth as compared with keratinocytes. While the latter show a diploid phenotype with 36 chromosomes, as reported for *Mastomys coucha* [25], explanted Kera5 cells were already found to be polyploid at passage 8 (n = 100, with a range of 92 to 111 chromosomes). Although cells in passage 163 were found to contain a smaller set of chromosomes (n = 83, with a range of 78 to 86), they still showed chromosomal instability, frequently manifested as chromatid breaks, acentric fragments and rearrangements (Fig 3B).

### Analysis of the p53 status in the Kera5 cell line

**Fig 4:**
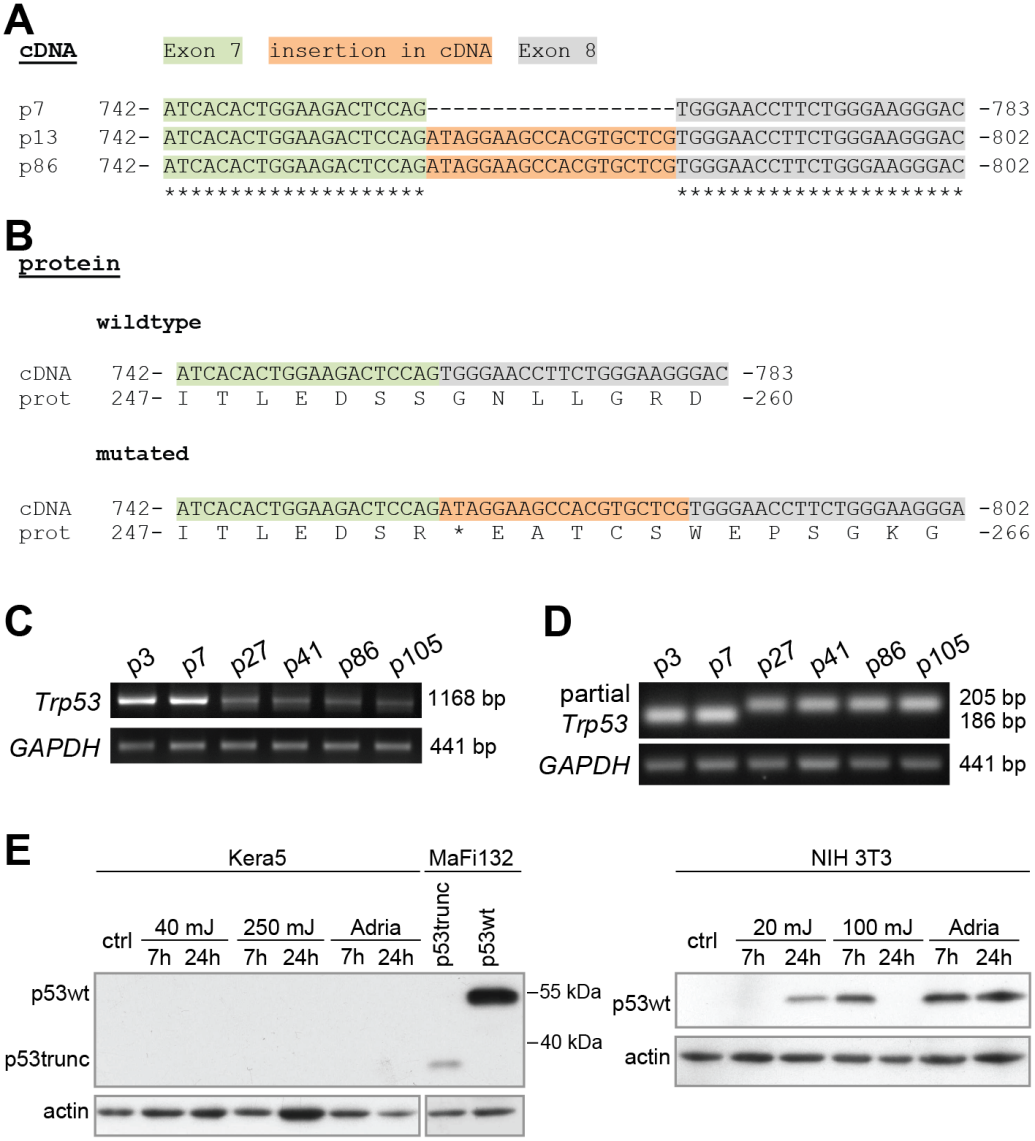
p53 cDNA sequence and transcriptional analyses. **(A)** The relevant nucleotides of the mutated p53 cDNA are shown. At passage 13, an insertion of 19 nt is detectable in the cDNA of p53 (exon 7: green, exon 8: grey, insertion: orange). **(B)** Comparison of translated wildtype and mutant sequences derived from the cDNA. The insertion leads to a premature stop codon (*) that results in a truncated form of p53. **(C)** Semi-quantitative RT-PCR of *Trp53* at different passages. *GAPDH* was used as reference gene. **(D)** Semi-quantitative RT-PCR to detect the insertion within the p53 cDNA, resulting in a slower-migrating PCR product at higher passages (p27-p105). *GAPDH* was used as reference gene. **(E)** Western blotting. Left panel: lack of p53 expression in Kera5. Kera5 (p155) were exposed to UVB (40 or 250 mJ/cm^2^) or treated with adriamycin (1.0 μg/ml) and harvested as indicated. 50 μg protein/lane were loaded. To control the specificity of the p53 antibody, MaFi132 were transiently transfected with pPK-CMV-E3 containing the cDNA for wildtype (p53wt) or the truncated (p53trunc)) form of p53. Since only 25 μg of protein were loaded for transfected MaFi132, a longer exposure time was chosen for actin. Right panel: NIH 3T3 cells were used as reference for stabilization of p53 after cells damage with UV or Adriamycin, respectively. NIH 3T3 cells were exposed to UVB (20 or 100 mJ/cm^2^) or treated with Adriamycin (0.75 μg/ml) and harvested as indicated. 50 μg protein/lane were loaded. After low dose UVB, p53 stabilization occurs slowly, whereas after high dose UVB the stabilization is fast, but transient. Adriamycin permanently stabilizes p53 already after 7h. Actin was used as loading control.

Since *TP53* is frequently mutated in cancer and also in immortalized cell lines [26, 27], we amplified and sequenced the corresponding cDNA obtained from the keratinocytes. Notably, already at passage 13, a 19 nt fragment inserted within the coding sequence of p53 could be detected that was not present in cDNA of freshly isolated *Mastomys*-derived keratinocytes (passage 0 until passage 7). This insertion leads to a frameshift and the appearance of a premature stop codon (Fig 4A), resulting in a truncated form of the p53 protein (Fig 4B). Although still expressed at higher passages, the splicing mutation apparently affects the steady-state level of the p53 mRNA, since less mRNA could be amplified (Fig 4C). Moreover, at higher passages all detectable p53-specific transcripts harbor this insertion, as directly visualized by a migration shift using primers in RT-PCR that were binding to sequences in the adjacent exons 7/8 (Fig 4D). Since the insertion leads to the appearance of a premature stop codon (Fig 4B), no p53 protein could be detected by Western blotting even after treatment with UV or adriamycin, respectively (Fig 4E, left panel) [28–30]. Confirming the experimental settings of cell damage as a result of these treatments, wildtype p53 protein was stabilized in NIH 3T3 cells (Fig 4E, right panel). Additionally, as a positive control for the specificity of the antibody against *Mastomys coucha* p53, MaFi132 cells were transiently transfected with expression vectors encoding either the wildtype or the truncated form of p53, which were both detectable. This indicates that the absence of mutated p53 in higher passage Kera5 cells was not due to the lack of cross-reactivity.

**Fig 5:**
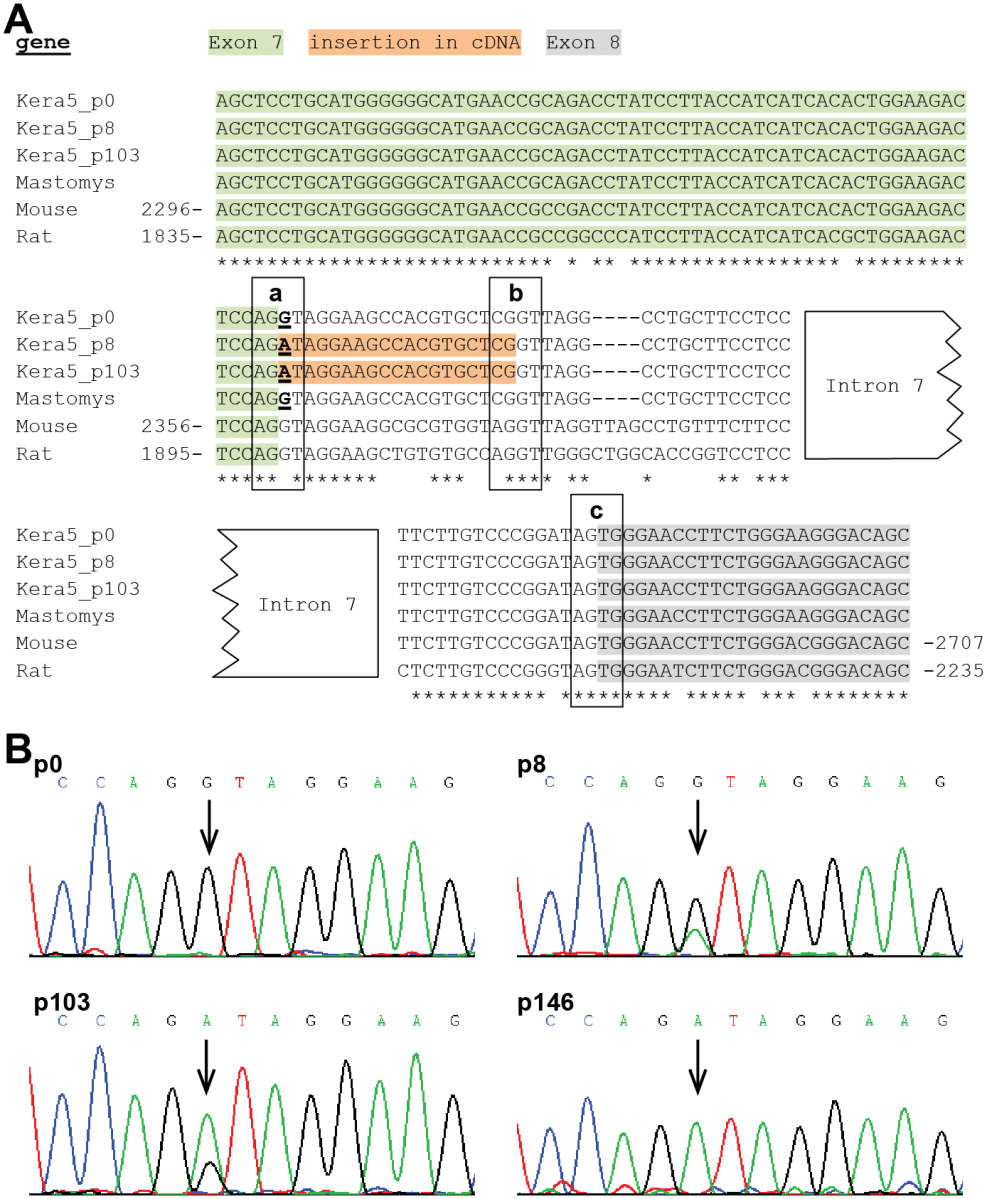
Sequencing of the *Mastomys* p53 gene (*Trp53*) and partial alignment with mouse and rat sequences. **(A)** Sequencing of *Trp53* in Kera5 (passages 8 and 103) shows a G>A transition at the first position of intron 7 (underlined). Freshly isolated primary keratinocytes (Kera5, p0) as well as five individual *Mastomys* samples do not harbor this mutation and are similar to murine and rat sequences at this position. Numbers refer to the sequences of murine *Trp53* and rat *Tp53*. For intron 7, only 5´-start and 3´-ends are shown. Exon 7: green, exon 8: grey, insertion: orange, frames: splicing signals; “a” indicates the original splicing donor, “b” alternative splicing donor signal in intron 7, “c”: splicing acceptor. **(B)** Sequencing chromatograms of *Trp53* reveal the G>A transition in a subpopulation of Kera5 cells at p8 which is not present at p0. A switch of the major peak from G to A from p8 to p103 occurs, suggesting the outgrowth of a single cell colony. At p146 only the A peak is left, revealing homozygosity. The arrows indicate the position of the mutation.

Comparison of the *Mastomys* p53 cDNA to its murine and rat homologs revealed that the position of the insertion in the coding sequence could be mapped to the junction of exon 7/8. In order to unravel the source of this insertion, intron 7 from *Trp53* was amplified by PCR from total genomic DNA using primers binding at the 3´-end of exon 7 and the 5´-start of exon 8. Total genomic DNA was obtained from five individual *Mastomys* and primary Kera5 (p0) or Kera5 at later passages. The alignment of murine, rat and *Mastomys* target regions of *Trp53* shows a high degree of conservation of exons 7 and 8 (Fig 5A). Also the 5´-end of the intron, corresponding to the splicing donor sequence, is perfectly conserved in *Mastomys* samples and the freshly explanted keratinocytes (Kera5, p0). However, in Kera5 at later passages (8+), a subpopulation of cells shows a G>A transition at the first nucleotide of intron 7 (Fig 5A). Due to this point mutation, the splicing signal (indicated as frame “a” in Fig 5A) is changed from AGGT to AGAT, making it a weaker splicing donor [31] and favoring the use of a cryptic splicing donor 19 nt downstream (indicated as frame “b”). Therefore the insertion observed in the cDNA represents the result of an alternative splicing event, leading to an mRNA incapable to translate a functional p53 protein. Furthermore, this mutation cannot be found in genomic DNA obtained from primary keratinocytes (p0), but appears in the sequencing chromatogram at passage 8 (p8), becomes more predominant at p103 and represents the only peak at p146 (Fig 5B).

### Immortalized Kera5 are capable of differentiation

**Fig 6:**
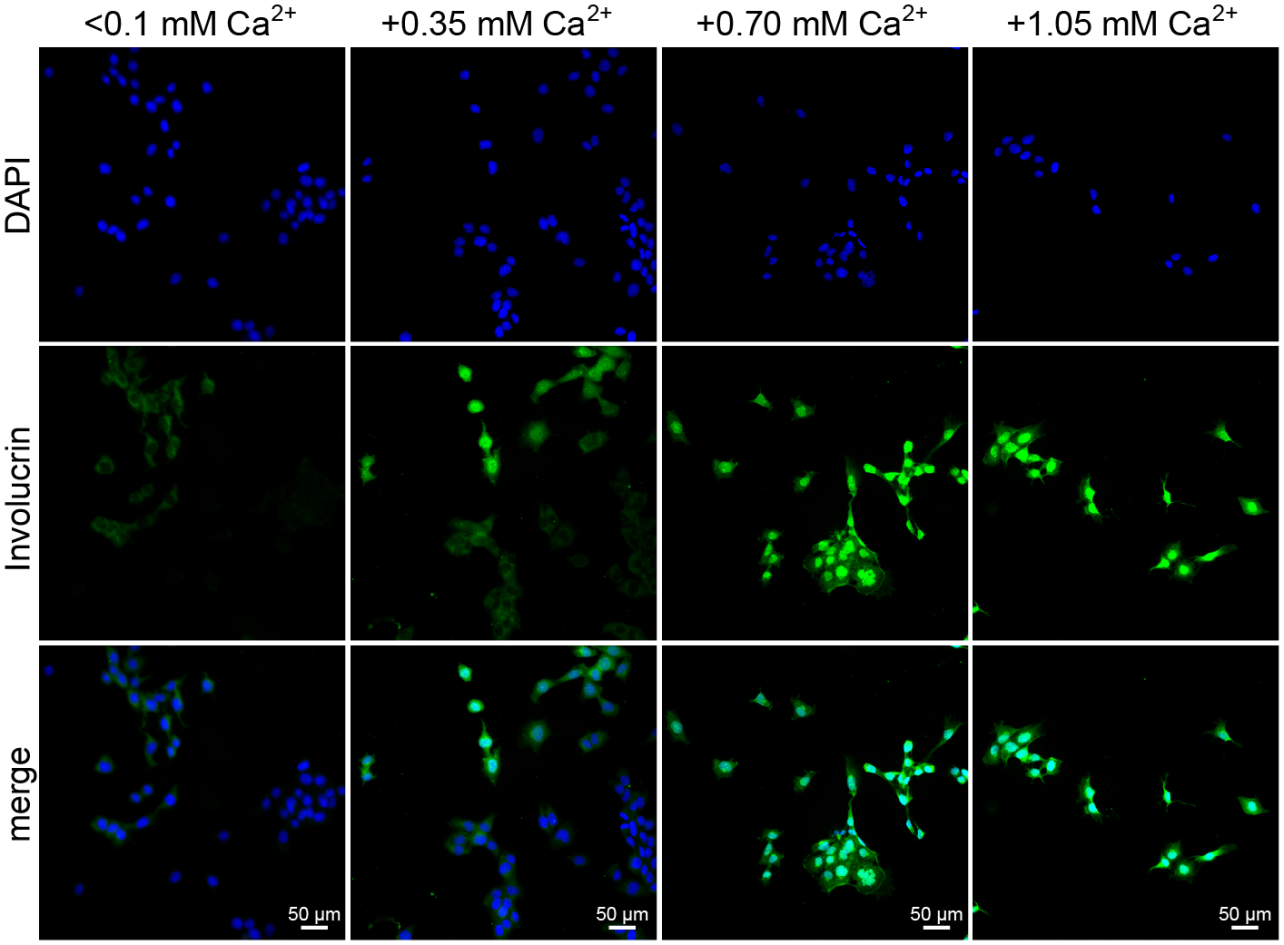
Calcium-induced differentiation of Kera5. Kera5 cells at passage 139 were grown on cover slips. After 24 h, the dKSFM (<0.1 mM Ca^2+^) was additionally supplemented with 0.35 mM, 0.7 mM or 1.05 mM Ca^2+^ to induce differentiation. After additional 24 h incubation, the cells were fixed with acetone, stained with an involucrin antibody and detected with an AlexaFluor488 secondary antibody, respectively. Green fluorescence indicates elevated involucrin expression in a dose-dependent manner (original magnification: 200x).

For certain experimental approaches it is mandatory that skin keratinocytes keep their ability to differentiate. This process can be monitored using certain markers, such as involucrin that becomes expressed [32, 33]. In order to analyze whether Kera5 at passage 139 are still capable of differentiation *in vitro*, the cells were incubated with increasing concentrations of calcium and subsequently stained for involucrin (Fig 6). While Kera5 cultured in low calcium medium (<0.1 mM Ca^2+^) only showed very weak involucrin expression, it is progressively increased after addition of 0.35 mM, 0.7 mM or 1.05 mM Ca^2+^, respectively.

## Discussion

In the present study, we established the first keratinocyte cell line (Kera5) derived from *Mastomys coucha*. The epidermal origin of Kera5 was confirmed by immunofluorescence specific for keratin 14 and by counterstaining against vimentin as mesenchymal marker (Fig 2). Kera5 already acquired a polyploid karyotype at passage p8 with an average of n = 100 chromosomes that were reduced to an average number of n = 83 in later passages, compared to the original chromosomal set of *Mastomys coucha* splenocytes (n = 36) [25]. Polyploidization as a cellular stress response when culturing primary cells is linked to chromosomal instability, accompanied with increased cell size (Fig 1) [21, 22], chromatid breaks and rearrangements (Fig 3). This seems to be in fact an early event during immortalization of murine and human cell lines [34–36] favoring the accumulation of clones with selective growth advantage. Between early and late passages, probably redundant chromosomes were lost, resulting in a more stabilized karyotype.

Polyploidy can arise in cells with loss or inactivation of p53 [37]. In fact, we identified a point mutation in intron 7 of *Trp53* that affects the splicing junction between exons 7 and 8, thereby leading to an alternative and frame-shifted splice variant (Fig 4A, 4B and 4D). Generally, splicing signals in human and murine pre-mRNAs are formed by the consensus sequence AGGU, whereby AG belongs to the 3´-end and GU to the 5´-end of the splicing donor/acceptor [31]. Exactly this signal can be found in mouse and rat as well as in five individual *Mastomys* samples and primary Kera5 cells (Fig 5A). Conversely, in Kera5 cells examined at passage 8 and later, the point mutation changed the donor signal to AGAU (Fig 5A, indicated as frame a), which is apparently no longer recognized by the splicing machinery. Instead, a CGGU sequence 19 nt downstream is used as a donor (Fig 5A, indicated as frame b), which constitutes a stronger signal than AGAU [31]. The use of alternative 5´-splicing sites accounts for 18% of alternative splicing in human and mice [31]. Due to the alternative 5´-splicing site, the first 19 nt of intron 7 are not spliced out and are still present in the processed mRNA leading to a frameshift, a stop codon and a truncated protein. Translation of this mRNA would result in a p53 isoform with 253 instead of 386 amino acids, lacking one third of its C-terminal sequence partly including the DNA-binding domain as well as both the complete nuclear-localization and oligomerization domains [38]. Consequently, in contrast to wildtype p53, the truncated form is not functional with respect of its transactivating activity, necessary to induce downstream target genes (see S1 Fig)[39]. It is therefore tempting to speculate that this mutation and the resulting alternative splicing provide a selective advantage, strong enough to facilitate growth advantage and cell immortalization. This assumption is supported by the sequencing chromatograms (Fig 5B) showing a switch from one G peak at p0 to a double peak at p8 and p103 and an accumulation of a single A peak at p146, characteristic for Kera5 cells maintained in culture for more than 175 passages up to date.

Notably, Kera5 cells at higher passage do not express any detectable p53 protein even after stimulation with UVB or adriamycin (Fig 4E). This is consistent with the notion that the majority of the detected p53 transcripts shows this insertion, leading to a premature stop codon at position 254 and protein degradation. Hence, the absence of p53 was not due to a lack of cross-reactivity of the antibody, since both wildtype and truncated p53 could be detected after transfecting the corresponding cDNA in MaFi132 cells. Notably, in the transfected positive controls, the cloned truncated p53 was detected in much lower amounts than wildtype p53. Considering this observation, it is improbable that this is due to a lower binding affinity, since the Pab240 antibody was raised against the N-terminal part of the DNA-binding domain (residues 156–214) of human p53, which shares 94% of homology to *Mastomys* p53 and is completely present in the truncated form. Thus, the absence of detectable p53 levels in Kera5 might be due to lower expression levels or a higher instability of the protein leading to degradation even after cell damage upon UV or adriamycin treatment. Alternatively, mutations in splicing junctions or premature stop codons can also affect the half-life of the corresponding mRNA or even result in its complete degradation, a phenomenon known as nonsense mediated decay [40, 41]. This may account for the reduced p53 steady-state mRNA levels at higher passages (Fig 4C).

Increasing calcium concentrations in stratifying epidermal layers induce keratinocyte differentiation, leading to a change in the expression pattern of certain markers such as keratins and involucrin [42]. Especially papillomaviruses are well-adapted to this mechanism and their replication depends on the differentiation status of the host cell [43]. As shown in Fig 6, despite of immortalization, Kera5 cells still show differentiation upon calcium treatment, as demonstrated by an increased expression of involucrin in a dose-dependent manner.

In summary, Kera5 is the first immortalized skin keratinocyte cell line derived from the animal model *Mastomys coucha*. The cells will be utilized in further studies to dissect the viral oncoprotein-cell interactome and to better understand the role of papillomaviruses in the development of non-melanoma skin cancer. Moreover, since this rodent is also used as model by other researchers with a focus on infectious agents [44, 45], the availability of such a cell line will be a beneficial contribution to these fields.

## Supporting Information

S1 Fig Functional analysis of p53 in a transactivation reporter assay. H1299 cells were co-transfected with 50 ng of expression vectors coding for wildtype (pPK-p53wt) or truncated (pPK-p53trunc) p53, 400 ng pG13-luc coding for firefly luciferase under control of p53-binding sites of the p21 promoter and 100 ng pRL-TATA coding for TATA-box controlled *Renilla* luciferase for normalization of the signals. Transfections were performed in duplicates. Cells were lysed 24 h after transfection and 20 μl of the lysates measured in a Dual-Luciferase^®^ Assay. Light units of the truncated p53 protein were normalized to wildtype p53 (n = 4, p<0.0001, t-test).
(TIF)

S1 Table Primers used in this study. (DOCX)
