# Supplementary figures and images for: Establishment of an Immortalized Skin Keratinocyte Cell Line Derived from the Animal Model Mastomys coucha
Source: PLoS One. 2016 Aug 17;11(8):e0161283. doi: 10.1371/journal.pone.0161283 (PMC4988767; doi:10.1371/journal.pone.0161283)

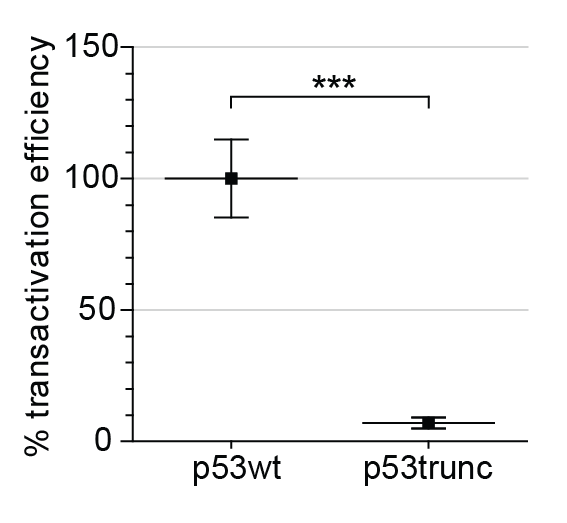

Supplement: S1 Fig — H1299 cells were co-transfected with 50 ng of expression vectors coding for wildtype (pPK-p53wt) or truncated (pPK-p53trunc) p53, 400 ng pG13-luc coding for firefly luciferase under control of p53-binding sites of the p21 promoter and 100 ng pRL-TATA coding for TATA-box controlled Renilla luciferase for normalization of the signals. Transfections were performed in duplicates. Cells were lysed 24 h after transfection and 20 μl of the lysates measured in a Dual-Luciferase® Assay. Light units of the truncated p53 protein were normalized to wildtype p53 (n = 4, p<0.0001, t-test). (TIF) [file pone.0161283.s001.tif]
