# Supplementary material for: Establishment of an Immortalized Skin Keratinocyte Cell Line Derived from the Animal Model Mastomys coucha
Source: PLoS One. 2016 Aug 17;11(8):e0161283. doi: 10.1371/journal.pone.0161283 (PMC4988767; doi:10.1371/journal.pone.0161283)
Supplement: S1 Table — (DOCX) [file pone.0161283.s002.docx]

## Supplemental items

## S1 Table. Primers used in this study.

| **Amplification of partial Trp53** |  |
| --- | --- |
| for | 5’-CGGCTCTGACTATACCACCATCC-3’ |
| rev | 5’-TTCCTCTGTACGACGGTCTCTCC-3’ |
| **Sequencing of partial Trp53** |  |
| rev | 5’-TTCCTCTGTACGACGGTCTCTCC-3’ |
| **Amplification of p53 cDNA** |  |
| for | 5’-TGACAGCCATGGAGTATTCGG-3’ |
| rev | 5’-CTGAGTCAGGCCCCACTTTC-3’ |
| **Sequencing of p53 cDNA** |  |
| for | 5’-TATGAGCCACCCGAGG-3’ |
| rev | 5’-GTCTTCCAGTGTGATGATGG-3’ |
| **Amplification of partial p53 cDNA** |  |
| for | 5’-CGGCTCTGACTATACCACCATCC-3’ |
| rev | 5’-TTCCTCTGTACGACGGTCTCTCC-3’ |
| **Cloning of p53wt** |  |
| for | 5’-TTTTGAATTCGCCATGACAGCCATG -3’ |
| rev | 5’-TTTTGGATCCGTCTGAGTCAGGCCCCAC-3’ |
| **Cloning of p53mut** |  |
| for | 5’-TTTTGAATTCGCCATGACAGCCATG-3’ |
| rev | 5’-ATATGGATCCTCTGGAGTCTTCCAGTGTGAT-3’ |
| **GAPDH reference** |  |
| for | 5’-CTTCATTGACCTCAACTACATGGTC-3’ |
| rev | 5’-GCAGTGATGGCATGGACTGTG-3’ |
